# Extremely low-frequency electromagnetic field (ELF-EMF) enhances mitochondrial energy production in NARP cybrids

**DOI:** 10.1038/s41598-025-10536-7

**Published:** 2025-07-08

**Authors:** Mikako Ito, Zhizhou Huang, Kosaku Nomura, Masaki Teranishi, Fei Zhao, Hiroyuki Mino, Makoto Yoneda, Masashi Tanaka, Kinji Ohno

**Affiliations:** 1 Division of Neurogenetics, Center for Neurological Diseases and Cancer, Nagoya University Graduate School of Medicine, Nagoya, Japan; 2 Division of Material Science (Physics), Graduate School of Science, Nagoya University, Nagoya, Japan; 3 Graduate School of Health and Human Life Sciences, Fukui Prefectural University, Fukui, Japan; 4 Department of Neurology, Juntendo University Graduate School of Medicine, Tokyo, Japan; 5 Graduate School of Nutritional Sciences, Nagoya University of Arts and Sciences, Nisshin, Japan

**Keywords:** Neuropathy, ataxia, retinitis pigmentosa (NARP) syndrome, Extremely low-frequency electromagnetic field (ELF-EMF), Mitochondrial DNA, Mitophagy, Mitochondrial biogenesis, And mitohormesis, Neuromuscular disease, Spinocerebellar ataxia

## Abstract

A mutation (m.8993T > G) in *MT-ATP6* in mitochondrial DNA (mtDNA) causes the neuropathy, ataxia, retinitis pigmentosa (NARP) syndrome by impairing mitochondrial energy production. Extremely low-frequency electromagnetic field (ELF-EMF) suppresses mitochondrial oxidative phosphorylation (OXPHOS) Complex II and induces mitohormetic activation of mitochondrial OXPHOS activities. We examined the effects of ELF-EMF on normal cybrids carrying 100% wild-type mtDNA (2SA cybrids) and NARP cybrids carrying 40% wild-type and 60% mutant mtDNA (NARP3-2 cybrids). We found that ELF-EMF had no effect on the copy number of mtDNA either in 2SA or NARP3-2 cybrids, or the ratio of wild-type-to-mutant mtDNA in NARP3-2 cybrids. Instead, ELF-EMF increased the transcription of mtDNA and the transcription ratio of wild-type-to-mutant mtDNA in NARP3-2 cybrids. In addition, ELF-EMF increased the expression of mitochondrial OXPHOS proteins and the mitochondrial OXPHOS Complex V activity in NARP3-2 cybrids. ELF-EMF upregulated fission-promoting phosphorylation of DRP1, as well as the expression of fusion-promoting MFN1 and MFN2, in NARP3-2 cybrids. ELF-EMF also increased ATP production estimated by oxygen consumption rates (OCR) and by a biochemical assay in NARP3-2 cybrids. Hormetic activation of mitochondria by ELF-EMF is likely to be effective to ameliorate defective mitochondrial energy production in NARP and other mitochondrial diseases.

**Supplementary Information:**

The online version contains supplementary material available at 10.1038/s41598-025-10536-7.

## Introduction

Extremely low-frequency electromagnetic field (ELF-EMF) that is comprised of 4-ms pulses of 10 µT electromagnetic field and is repeatedly modulated from 1 to 8 Hz in 8 s minimized the thermal hysteresis of modified Ringer’s solution^1^. We showed that ELF-EMF specifically suppressed mitochondrial oxidative phosphorylation (OXPHOS) Complex II and reduced the mitochondrial mass and the mitochondrial membrane potential in mouse hepatocyte-derived AML12 cells^2^. The exact target molecule or moiety of ELF-EMF in OXPHOS Complex II remains undetermined. ELF-EMF induced mitophagy by suppressing OXPHOS Complex II and subsequently upregulated PGC-1α and mitochondrial biogenesis^2^. ELF-EMF, the condition of which was identical to that explained above, was effective for a mouse model of depression by enhancing mitochondrial energy production^3^as well as for patients with treatment-resistant depression^4^. Similarly, ELF-EMF (1 to 20 µT at 1 to 80 Hz) alleviated symptoms of children with autism spectrum disorder^5^. In addition, ELF-EMF (1 µT at 8/6 Hz) decreased blood pressure in patients with hypertension^6,7^. We also showed that ELF-EMF induced heat shock responses in AML12 and HEK293 cells, which was likely to be a secondary response associated with mitohormesis^8^.

The neuropathy, ataxia, retinitis pigmentosa (NARP) syndrome is caused by heteroplasmic pathogenic variants in *MT-ATP6* in mitochondrial DNA (mtDNA) that encodes subunit 6 of mitochondrial H^+^-ATPase or mitochondrial OXPHOS Complex V. Six variants have been reported in *MT-ATP6* in patients with NARP: m.8993T > G (p.Leu156Arg), m.8993T > C (p.Leu156Pro), m.8839G > C (p.Ala105Pro), m.8989G > C (p.Ala155Pro), m.8618insT (p.Thr33Hisfs*31), and m.9185T > C (p.Leu220Pro)^9^. Among them, m.8993T > G constitutes about 50% of variants in patients with NARP^9^. Pathogenic variants in *MT-ATP6* also cause mitochondrial Leigh syndrome (MILS) when the ratio of mutant mtDNA is high^10^. In contrast to NARP, MILS is also caused by pathogenic variants in mitochondrial tRNAs (*MT-TV*,* MT-TK*,* MT-TW*, and *MT-TL1*)^10^. Two lines of *trans*-mitochondrial hybrid clones with m.8993T > G (NARP3-1 carrying 98% mutant mtDNA and NARP3-2 carrying 60% mutant mtDNA) were generated by fusing patient’s platelets with human osteosarcoma 143B cells lacking mtDNA (ρ^o^206 cells)^11,12^. The cybrids are able to disclose the effects of mutant mtDNA without being affected by variants on nuclear DNA (nDNA), and also the effects of variable heteroplasmic ratio of mutant mtDNA^13^.

Leu156, the mutant residue of m.8993T > G, is located on helix H5 and is buried in the core of ATP6^14^. p.Leu156Arg by m.8993T > G damages the interaction between subunit helices H5 and H6 because of a larger volume of arginine compared to leucine and of a charged residue introduced into a cluster of hydrophobic residues^14^. In addition, the analyses of the effects of m.8993T > G in yeasts, platelets, lymphocytes, muscle tissue, fibroblasts, and cybrids revealed that the enzymatic activities of both ATP synthase and ATP hydrolysis, as well as ATP production estimated by oxygen consumption rates (OCR), were decreased in most models cell^15^. Thus, the pathomechanisms of m.8993T > G are likely to be due to a combination of (i) defective proton transport across Fo, (ii) failure to couple phosphorylation of ADP on F1 to proton flow, and (iii) alteration of the holoenzyme assembly and stability^15^.

Although ELF-EMF enhances mitochondrial OXPHOS activities by inducing mitohormesis, the effects of ELF-EMF on mitochondrial diseases have not been investigated to date. As expected, we found that ELF-EMF ameliorated defective energy production in NARP cybrids by inducing mitohormesis.

## Results

### ELF-EMF increases the expression of mitochondrial OXPHOS complexes in NARP3-2 cybrids

**Fig. 1:**
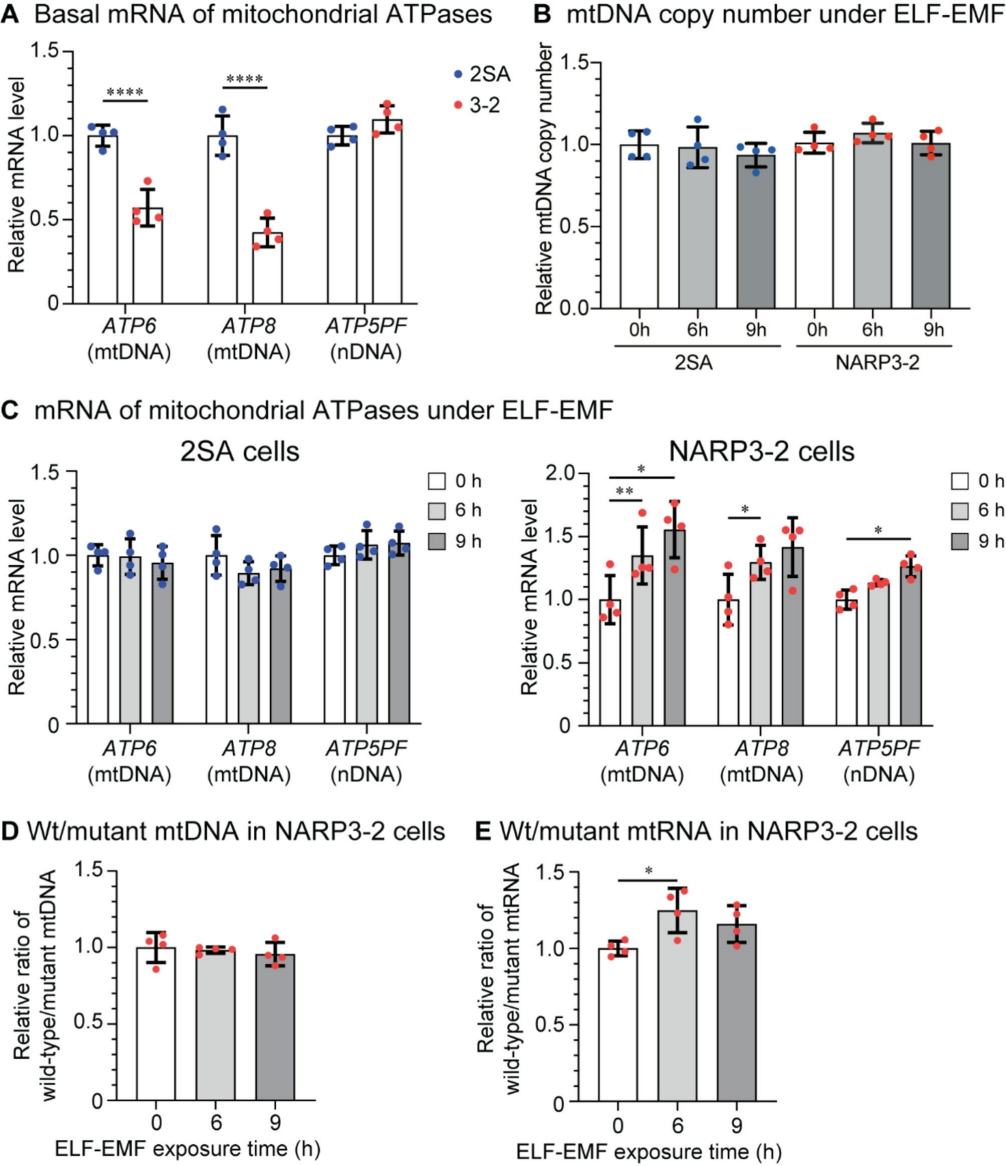
ELF-EMF upregulates the mitochondrial OXPHOS Complexes and the Complex V activity in NARP3-2 cybrids. **(A)** Basal mRNA of mitochondrial DNA-encoded *ATP6* and *ATP8*, and nDNA-encoded *ATP5* in 2SA and NARP3-2 cybrids without ELF-EMF. **(B)** mtDNA copy numbers under ELF-EMF exposure. **(C)** mRNA levels of mitochondrial ATPases in 2SA and NARP3-2 cybrids under ELF-EMF exposure. **(D**,** E)** Relative ratios of wild-type to mutant mtDNA **(D)** and mtRNA **(E)** in NARP3-2 cybrids under ELF-EMF exposure. **(B**,** D)** mtDNA copy numbers were normalized for the nDNA-encoded *YWHAZ* gene and also for the value at 0 h of 2SA cybrids. **(A**,** C**,** E)** mtRNA levels were normalized to *YWHAZ* mRNA and also for the value at 0 h. Mean and SD are indicated. *P < 0.05, **P < 0.01, ***P < 0.005, and ****P < 0.0001 by ordinary two-way ANOVA **(A)**, repeated measures two-way ANOVA **(C)**, or one-way ANOVA **(B**,** D**,** E)** followed by Dunnett’s posthoc test.

As NARP3-2 cybrids carried a mutation in *MT-ATP6*, we first observed that mRNA levels of mtDNA-encoded *ATP6* and *ATP8* were decreased in NARP3-2 cybrids compared to normal 2SA cybrids (Fig. 1A). In contrast, mRNA levels of nDNA-encoded *ATP5PF* were similar between the two cybrids. We next examined whether ELF-EMF increased mtDNA copy numbers and found that ELF-EMF did not have such an effect in either 2SA or NARP3-2 cybrids (Fig. 1B). Instead, ELF-EMF increased mRNA levels of mitochondrial ATPases in NARP3-2 cybrids (Fig. 1C). We expected that ELF-EMF enhanced biogenesis of specific mitochondrial species with a high ratio of wild-type mtDNA and increased the ratio of wild-type-to-mutant mtDNA in NARP3-2 cybrids, but did not observe such an effect (Fig. 1D). Instead, ELF-EMF increased the ratio of wild-type-to-mutant mitochondrial RNA (mtRNA) (Fig. 1E). Taken together, ELF-EMF had no effect on mtDNA copy numbers or the ratio of wild-type-to-mutant mtDNA, but enhanced transcription of wild-type mtDNA more than mutant mtDNA.

### ELF-EMF upregulates mitochondrial OXPHOS complex proteins in NARP3-2 cybrids

**Fig. 2:**
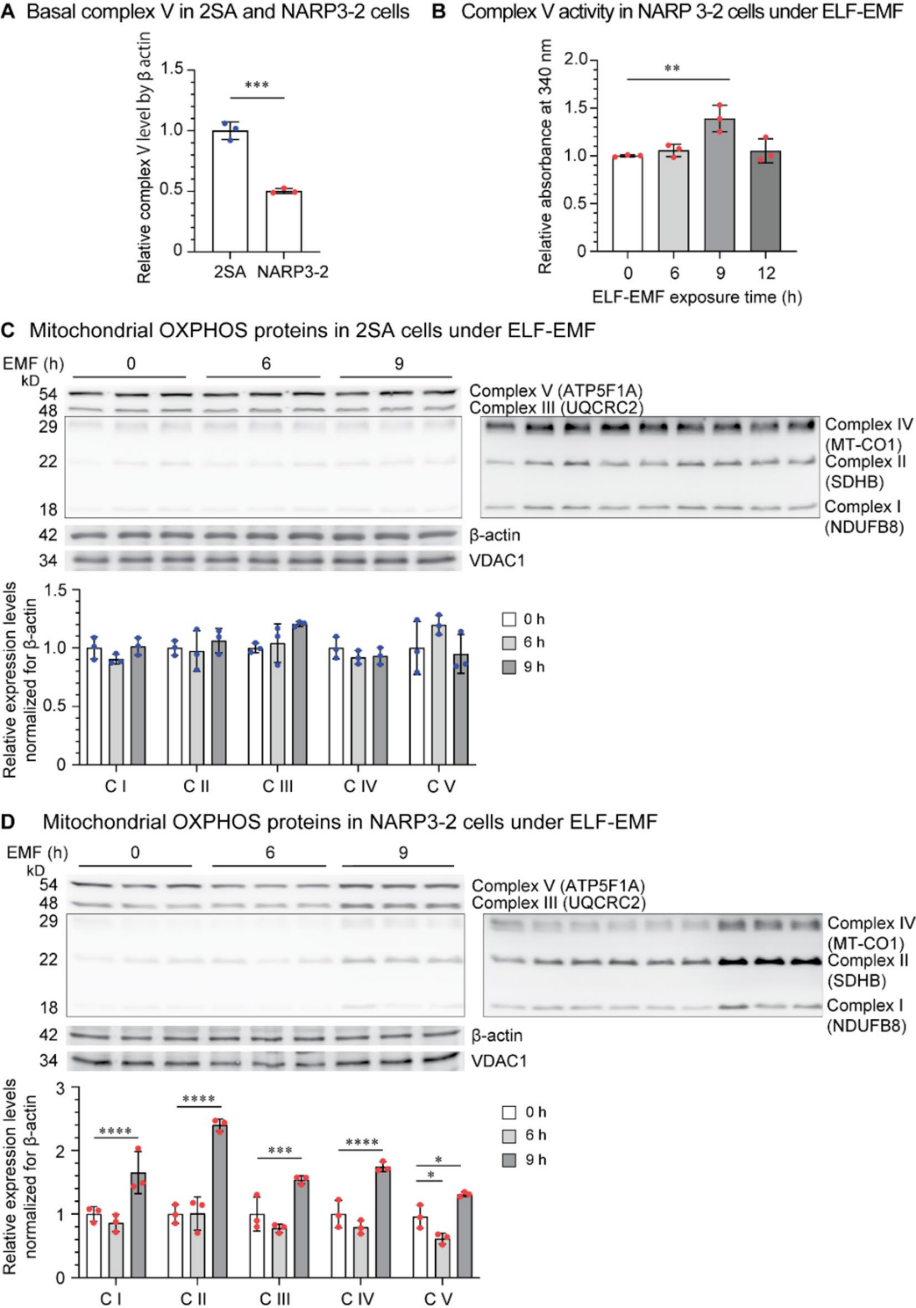
ELF-EMF upregulates the mitochondrial OXPHOS Complexes and the Complex V activity in NARP3-2 cybrids. **(A)** Basal expression of ATP5F1A in Complex V in 2SA and NARP3-2 cybrids without ELF-EMF. **(B)** Mitochondrial Complex V activity under ELF-EMF in NARP3-2 cybrids. **(C**,** D)** Western blotting and quantification of representative proteins constituting mitochondrial OXPHOS Complexes in 2SA **(C)** and NARP3-2 **(D)** cybrids under ELF-EMF. Images with longer exposure times are indicated in the right panels. C, mitochondrial OXPHOS Complex. Mean and SD are indicated. *P < 0.05, **P < 0.01, ***P < 0.005, and ****P < 0.0001 by unpaired Student’s *t*-test **(A)**, one-way ANOVA **(B)** or repeated-measures two-way ANOVA **(C**,** D)** followed by Dunnett’s posthoc test.

The basal protein expression of ATP5F1A in Complex V was lower in NARP3-2 cybrids compared to 2SA cybrids (Fig. 2A). ELF-EMF exposure for 9 h increased the activity of mitochondrial OXPHOS Complex V in NARP3-2 cybrids (Fig. 2B). Similarly, Western blotting of representative proteins in five mitochondrial OXPHOS Complexes showed that mitochondrial OXPHOS Complex proteins tended to be decreased at 6 h after starting ELF-EMF exposure, but were increased at 9 h in NARP3-2 cybrids (Fig. 2D). However, these effects were not observed in 2SA cybrids (Fig. 2C). The biphasic effects of ELF-EMF on mitochondrial OXPHOS Complex proteins in NARP3-2 cybrids are consistent with our previous observations that ELF-EMF induced mitophagy that was followed by hormetic activation of mitochondrial biogenesis in mouse hepatocyte-derived AML12 cells^2^.

### ELF-EMF upregulates mitochondrial fission/fusion proteins in NARP3-2 cybrids

**Fig. 3:**
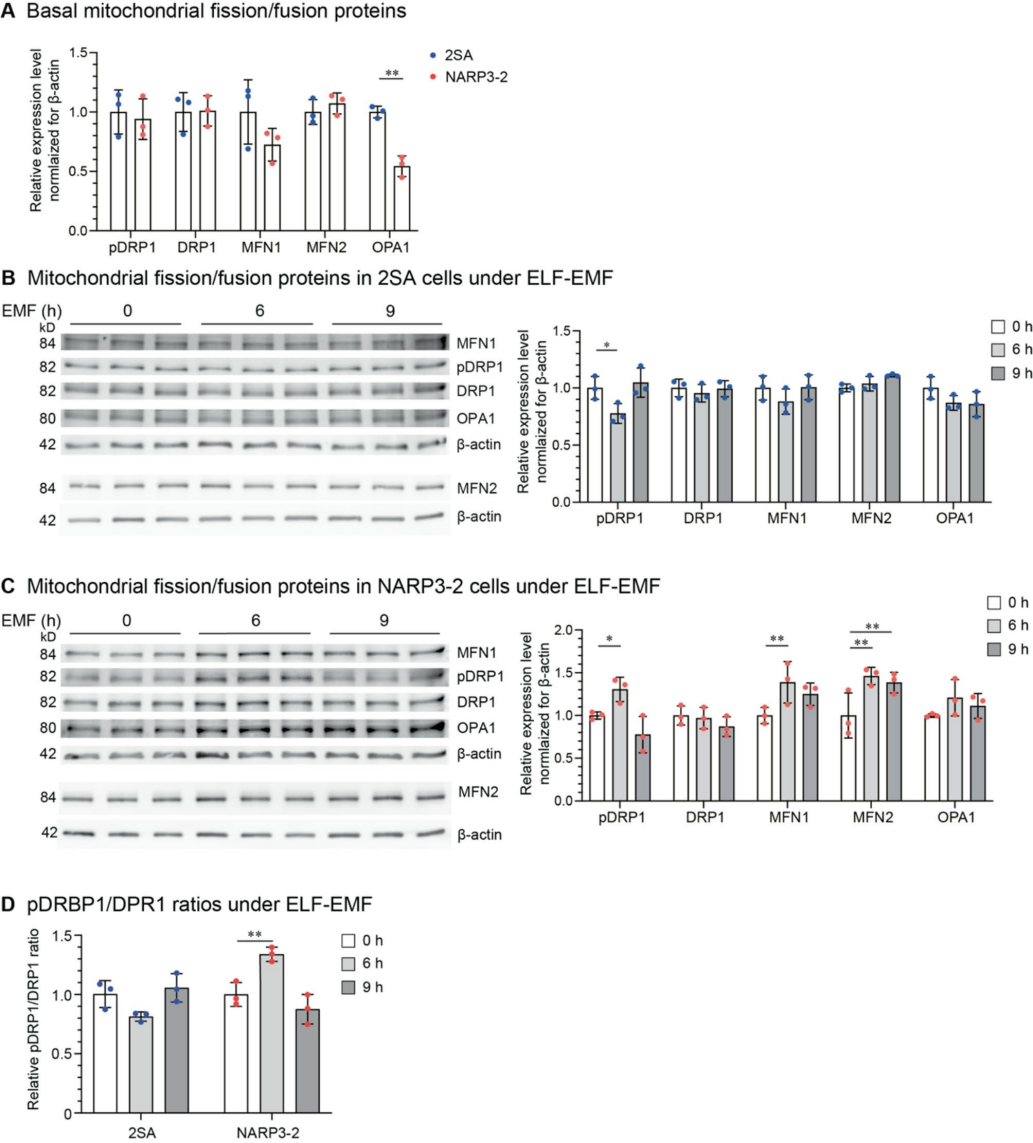
ELF-EMF upregulates mitochondrial fission/fusion proteins in NARP3-2 cybrids. **(A)** Basal expression of mitochondrial fission/fusion proteins in 2SA and NARP3-2 cybrids without ELF-EMF. **(B**,** C**,** D)** Mitochondrial fission/fusion proteins in 2SA **(B)** and NARP3-2 **(C)** cybrids under ELF-EMF exposure. Mean and SD are indicated. *P < 0.05, **P < 0.01, ***P < 0.005, and ****P < 0.0001 by ordinary two-way ANOVA **(A)** and repeated-measures two-way ANOVA **(B**,** C**,** D)** followed by Dunnett’s posthoc test.

We next observed that mitochondrial fusion proteins, mitofusin 1 (MFN1) and mitochondrial dynamin-like GTPase (OPA1) were lower in NARP3-2 cybrids compared to 2SA cybrids. In contrast, neither the expression level of the fission protein DRP1 nor the phosphorylation at Ser616 of DRP1, which stimulates mitochondrial fission during mitosis^16^, was different between 2SA and NARP3-2 cybrids (Fig. 3A). Similarly, mitofusin 2 (MFN2) was not different between 2SA and NARP3-2 cybrids. MFN1 and MFN2 function complementarily to maintain a balance between mitochondrial fusion and fission, and MFN2 is important for adaptation to mitochondrial stress and subsequently for mitochondrial quality control^17^. We found that ELF-EMF suppressed the phosphorylation at Ser616 of DRP1 in 2SA cybrids at 6 h, whereas ELF-EMF upregulated it in NARP3-2 cybrids at 6 h (Fig. 3BCD). ELF-EMF additionally upregulated the expression of MFN1 and MFN2 in NARP3-2 cybrids at 6 h (Fig. 3 C). Taken together, ELF-EMF was likely to activate both mitochondrial fission (pDRP1) and fusion (MFN1, MFN2) in NARP3-2 cybrids.

### ELF-EMF upregulates ATP production in NARP3-2 cybrids

**Fig. 4:**
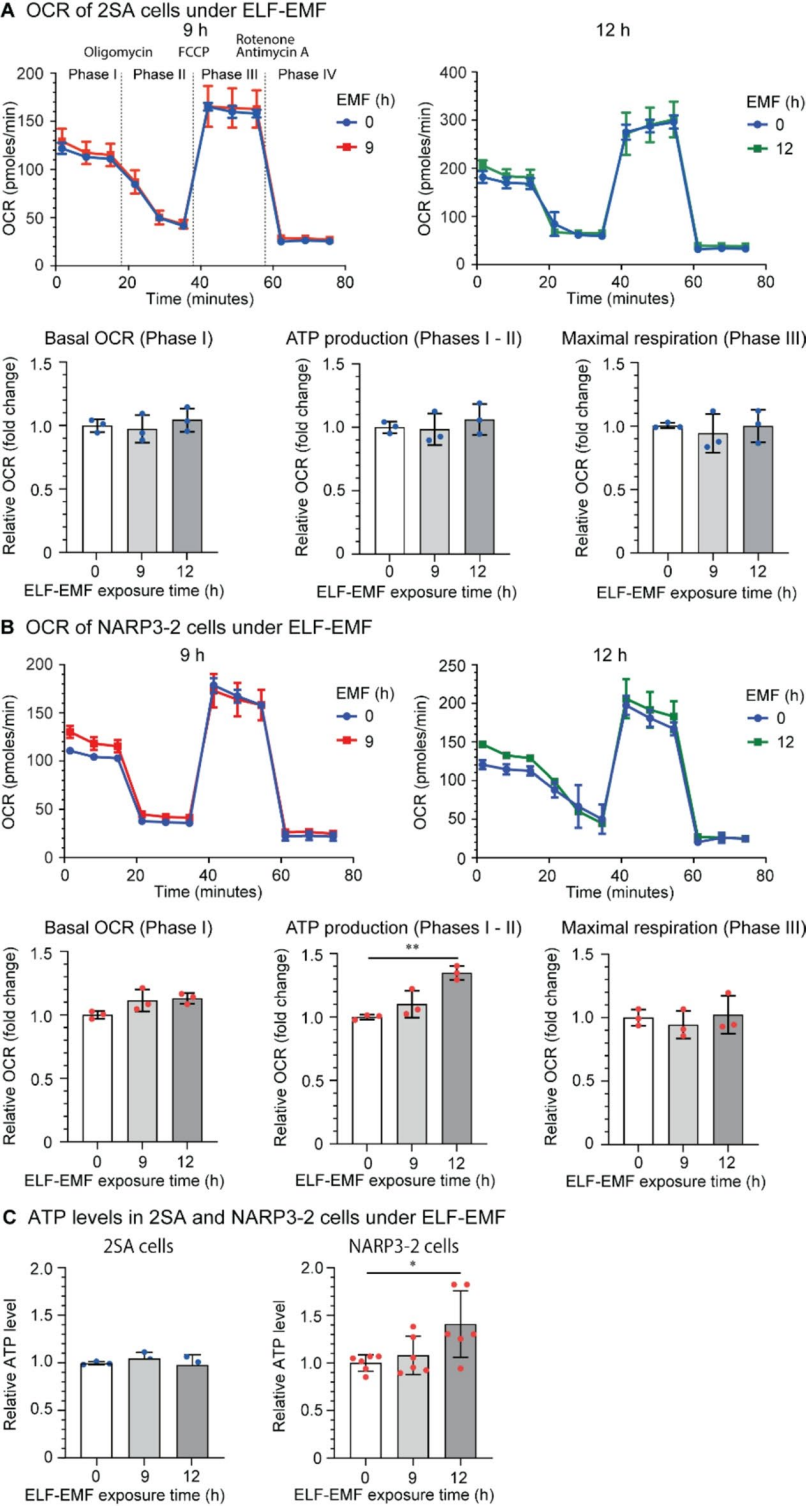
The effects of ELF-EMF on the oxygen consumption rates (OCR). OCR of 2SA **(A)** and NARP3-2 **(B)** cybrids under ELF-EMF. Chemical compounds to control mitochondrial respiration and OCR phases are indicted in the top left panel in **(A)**. **(C)** ATP levels in 2SA and NARP3-2 cybrids under ELF-EMF. Mean and SD are indicated. *P < 0.05, **P < 0.01, ***P < 0.005, and ****P < 0.0001 by one-way ANOVA followed by Dunnett’s posthoc test.

We next examined the effects of ELF-EMF on OCR. In OCR measurement, oligomycin inhibits mitochondrial ATPase, and mitochondrial ATP production is estimated by subtracting OCR before (phase I) and after (phase II) adding oligomycin. FCCP is a proton ionophore that cancels the proton gradient across the inner mitochondrial membrane. After adding FCCP, mitochondrial OXPHOS Complexes I-IV become fully operational to generate as much proton gradient as possible, and maximal mitochondrial respiration can be measured (phase III). We first observed that the OCR profiles of 2SA and NARP3-2 cybrids were essentially the same (Supplementary Fig. S1). Lack of the difference in OCR in NARP3-2 cybrids carrying 60% mutant mtDNA was in accordance with a previous study showing that the reduction of ATP production estimated by OCR correlated well with the ratio of mutant mtDNA in cybrids^18^. In 2SA cybrids, ELF-EMF had no effect on the basal OCR (phase I), ATP production (phases I-II), maximal respiration (phase III) (Fig. 4A), or the ATP level (Fig. 4C). In contrast, in NARP3-2 cybrids, ELF-EMF increased ATP production (phases I-II) by OCR measurement (Fig. 4B) and ATP level by a biochemical assay (Fig. 4C). Thus, ELF-EMF indeed enhanced ATP production by enhancing mitochondrial respiration in NARP3-2 cybrids.

## Discussion

**Fig. 5:**
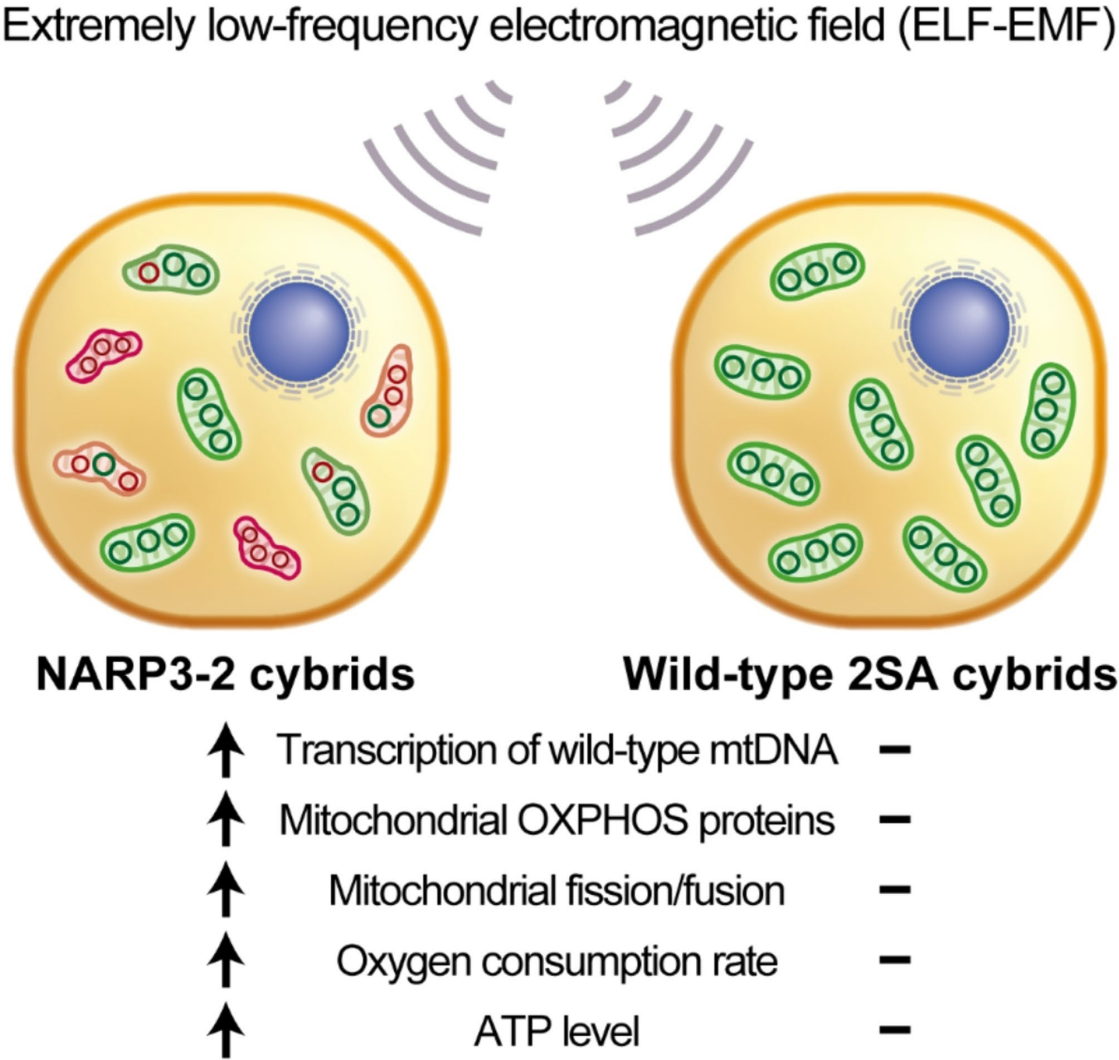
Schematic summary of the effects of ELF-EMF on NARP cybrids carrying 40% wild-type and 60% mutant mitochondrial DNA (mtDNA). ELF-EMF enhances mitochondrial energy production in NARP cybrids by increasing transcription of wild-type mtDNA, mitochondrial OXPHOS proteins, mitochondrial fission/fusion markers, oxygen consumption rate, and mitochondrial ATP production.

A summary of our studies is schematically shown in Fig. 5. We examined the effects of ELF-EMF on NARP3-2 cybrids carrying 40% wild-type and 60% mutant mtDNA. In NARP3-2 cybrids, ELF-EMF increased the transcription of wild-type mtDNA more than mutant mtDNA (Fig. 1E) without affecting mtDNA copy numbers (Fig. 1B) or the ratio of wild-type-to-mutant mtDNA (Fig. 1D). ELF-EMF also increased the protein expression levels of mitochondrial OXPHOS Complexes (Fig. 2D), mitochondrial fission/fusion markers (Fig. 3CD), and ATP production estimated by both OCR (Fig. 4B) and a biochemical assay (Fig. 4C) in NARP3-2 cybrids. As ELF-EMF was expected to have no direct effect on the enzyme kinetics of the mutant mitochondrial ATPase, the observed increase in ATP production was likely to be accounted for by hormetic induction of mitochondrial biogenesis, leading to consequent increases in mitochondrial OXPHOS Complex proteins.

We previously showed that ELF-EMF, the condition of which was identical to our current study (4-ms pulses of 10 µT electromagnetic field at 1–8 Hz in 8 s), induced mitochondrial biogenesis and significantly increased mitochondrial membrane potential by 14% in AML12 cells^2^. In 2SA cybrids, although mitochondrial OXPHOS proteins were increased by 4% on average, statistical significance was not observed in any proteins (Fig. 2C). In contrast, in NARP3-2 cybrids, ELF-EMF increased mitochondrial OXPHOS proteins by 73% on average (Fig. 2D). Lack of ostensive mitohormetic effects by ELF-EMF in control 2SA cybrids (Figs. 1C, 2C and 3BD, and 4AC) may represent sufficient basal mitochondrial energy production in 2SA cybrids compared to AML12 cells. We showed by OCR that ELF-EMF had no effects on OCR profiles in 2SA cybrids (Fig. 4A), but increased basal mitochondrial oxygen consumption (phases I-II) representing ATP production in NARP3-2 cybrids (Fig. 4B). The effects of ELF-EMF on NARP3-2 cybrids were consistent with our previous observations by OCR that ELF-EMF, the condition of which was identical to our current study, increased basal and maximal oxygen consumptions in AML12 and HEK293 cells^8^. We also showed by biochemical assay that ELF-EMF increased the ATP levels by 40.8% at 12 h in NARP3-2 cybrids (Fig. 4C). The increases were also in accordance with our previous observation that ELF-EMF increased the ATP levels by 61.8% and 31.4% in AML12 and HEK293 cells, respectively, in 3 h^8^. Extensive review of the effects of weak electromagnetic fields on reactive oxygen species (ROS) showed variable effects (increased in 11 cultured cells, decreased in 5 cultured cells, and unchanged in 14 cultured cells), but factors affecting the ROS production remained elusive^19^. Among them, the effects of electromagnetic fields on mitochondrial functions were analyzed in two studies. First, in contrast to the biphasic mitohormetic effects of ELF-EMF in our study, both stationary and alternating hypomagnetic fields (0.2–2.0 µT), which were 20 to 200 times lower than the geomagnetic fields, for three days decreased mitochondrial membrane potential in mouse primary myoblasts^20^. Second, again in contrast to our study, in human amniotic epithelial FL cells, ELF-EMF of 0.4 mT at 50 Hz for 1 h had no effect on mitochondrial membrane potential^21^.

ELF-EMF had oppositive effects on mitochondrial fission estimated by the phosphorylation at Ser616 of DRP1 in two cybrids: suppressed in 2SA cybrids and enhanced in NARP3-2 cybrids (Fig. 3BCD). The increase and decrease of DRP1 phosphorylation in 2SA and NARP3-2 cybrids, respectively, correlated with the presence and absence of the effects of ELF-EMF on mitochondrial biogenesis. This is in accordance with the notion that activated mitochondrial fission by DRP1 stimulates mitophagy and mitochondrial biogenesis^22–24^. However, in the prefrontal cortex in a mouse model of depression, ELF-EMF, the condition of which was identical to our current study, did not increase DRP1 phosphorylation at Ser616 but enhanced mitochondrial biogenesis^3^. Thus, the activation of mitochondrial fission is not a prerequisite for the induction of mitochondrial biogenesis by ELF-EMF. The increased expression of mitochondrial fusion protein, MFN1 and MFN2, by ELF-EMF in NARP3-2 cybrids (Fig. 3BC) may have similar effects on mitochondrial biogenesis^25^.

Therapeutic modalities have been sought for in cultured cells for NARP syndrome^15^. For example, mitochondrial ATP production was upregulated by a combination of α-ketoglutarate and aspartate^26^ and dimethyl-α-ketoglutarate, a membrane-permeable analog of α-ketoglutarate^27^ to boost mitochondrial substrate-level phosphorylation. We previously showed that therapeutic introduction of a gene encoding *Sma*I restriction enzyme that could digest only mutant (m.8993T > G) mtDNA increased mitochondrial membrane potentials in both NARP3-1 and NARP3-2 cybrids^28^. ELF-EMF is unique in that it does not use any artificial or natural compounds. The electromagnetic intensity of our protocol of ELF-EMF was 10 µT, which was much lower than the geomagnetic fields from 25 to 65 µT. As stated in the introduction, three clinical trials have been reported on ELF-EMF without adverse effects: hypertension at 1 µT^6,7^, treatment-resistant depression at 10 µT^4^, and autism spectrum disorder at 1-to-20 µT^5^. We expect that mitochondrial biogenesis by ELF-EMF is a promising therapeutic modality for patients with NARP and other mitochondrial diseases.

## Conclusions

We showed that ELF-EMF as weak as 10 µT provoked mitohormetic effects on NARP3-2 cybrids carrying 60% mutant mtDNA, but marginally on control 2SA cybrids. Mitohormetic effects on NARP3-2 cybrids included (i) the increased ratio of wild-type-to-mutant mtRNA, (ii) the increased expression of mitochondrial OXPHOS proteins, (iii) increased activity of the mitochondrial OXPHOS Complex V, (iv) upregulation of both mitochondrial fission and fusion markers, and (v) increased ATP production. We showed the effects of ELF-EMF on the model cells of NARP patients, but the actual effects of ELF-EMF on NARP patients need to be explored in the future.

## Methods

### Cybrid cell lines and their culture

The control 2SA^29–32^ and NARP3-2^28^ cybrid cell lines carrying 100% wild-type mtDNA and 40% wild-type:60% mutant mtDNA, respectively, were previously established by fusion of mtDNA-deficient ρ^o^206 cells generated from human 143B osteosarcoma cell line^13^ and enucleated myoblasts from patients with MELAS and NARP, respectively. In 2SA cybrids, only wild-type mtDNA in a heteroplasmic patient with MELAS was incorporated. The cybrids were cultured in high-glucose Dulbecco’s modified Eagle’s medium (DMEM) supplemented with 10% fetal bovine serum, 1 mM sodium pyruvate, and 0.4 mM uridine, and maintained under 5% CO_2_ at 37 °C.

### Exposure of cybrids to ELF-EMF

Cultured cybrids on a 6-well plate or 96-well plate were placed on the following ELF-EMF device in a humidified incubator at 37 °C. The ELF-EMF device was manufactured by the Technical Center of Nagoya University, Japan. The device was composed of a round coil (1 cm height, 10 cm inner diameter, and 10.7 cm outer diameter, 50 turns of copper wire of 0.29 mm diameter) that generated 100 mG (10 µT) electromagnetic field of 4 m pulse width with increasing frequencies of 1, 2, 3, 4, 5, 6, 7, and 8 Hz every second^2^. We previously showed that this protocol most efficiently induced mitophagy in AML12 cells^2^. Cell culture plates were placed directly above a magnetic field-generating coil for 6 and 9 h. Cell culture plates without ELF-EMF as a control were similarly placed on a coil, but the electricity was turned off. We confirmed the coil with the small and short current did not generate any detectable heat. To reduce the effects of an electromagnetic field generated by an incubator, the culture dish and the coil were sandwiched by two 5-mm thick copper plates, and were placed in a humidified incubator with 5% CO_2_ at 37 °C. The two copper plates also reduced the geomagnetic field, and all experiments were performed under ambient geomagnetic field. We previously showed that the coil exerted its effects irrespective of the direction of the geomagnetic field^2 ^but the coil was always placed in parallel to the ground surface in this study. The magnetic intensity by the coil was measured before and after each experiment.

### Analysis of mtDNA copy numbers and mtDNA transcription

DNA was extracted using DNeasy Blood & Tissue Kit (Qiagen) according to the manufacturer’s instructions. For qPCR of mtDNA and nDNA, primers were designed for *MT-ND6* and *YWHAZ*, respectively (Supplementary Table S1). The copy number of mtDNA was normalized to that of *YWHAZ*. RNA was extracted using RNeasy Mini Kit (Qiagen) according to the manufacturer’s instructions. Total RNA was reverse-transcribed using random hexamers (Thermo Fisher Scientific) and ReverTra Ace reverse transcriptase (Toyobo). Gene expressions were normalized for that of the *YWHAZ* gene, which was more stable than *B2M*,* RPLP0*,* ACTB*, or *GAPDH* under stress conditions or under cell proliferation and differentiation^33–35^. Primers for allele-specific qPCR and qRT-PCR to differentially amplify wild-type and m.8993T > G *MT-ATP6* are indicated in Supplementary Table S1. Primers for qRT-PCR of other genes are also indicated in Supplementary Table S1. Both qPCR and qRT-PCR were performed on LightCycler 480 II (Roche Diagnostics) using TB Green Premix Ex Taq II (Takara Bio). qPCR and qRT-PCR reactions for all primer pairs (Supplementary Table S1) were performed initially at 95 °C for 30 s followed by 40 cycles of 95 °C for 5 s and 60 °C for 20 s.

### Western blot analysis

Cultured cybrids were lysed in PLC buffer containing 50 mM HEPES (pH 7.0), 150 mM NaCl, 10% glycerol, 1% TritonX-100, 1.5 mM MgCl_2_, 1 mM EGTA, 100 mM NaF, 10 mM sodium pyrophosphate, 1 µg/µl aprotinin, 1 µg/µl leupeptin, 1 µg/µl pepstatin A, and 1 mM PMSF. The cell lysates were rotated at 4 °C for 20 min and centrifuged at 17,900 × *g* at 4 °C for 15 min. The supernatant was denatured at 37 °C for 1 h to analyze the mitochondrial OXPHOS Complex proteins or at 95 °C for 5 min to analyze other proteins in the sample buffer (62.5 mM Tris-HCl pH 6.8, 2% SDS, 10% glycerol, 0.005% bromophenol blue, and 2% 2-mercaptoethanol). The lysates were separated by Tris-SDS-PAGE on a 10 or 12% SDS-polyacrylamide gel. The samples were then transferred to a poly-vinylidene fluoride membrane (Immobilon-P, Millipore). Membranes were washed in Tris-buffered saline containing 0.05% Tween 20 (TBS-T) and blocked for 1 h in TBS-T with 5% skimmed milk. Membranes were incubated in the following primary antibody at 4 °C overnight: total OXPHOS rodent WB antibody cocktail (1:1000, ab110413, Abcam), anti-VDAC1 (1:3000, ab14734, Abcam), anti-β-actin (1:1000, sc-47778, Santa Cruz Biotechnology), anti-MFN1 (1:1000, 13798-1-AP, Proteintech), anti-MFN2 (1:1000, 121861-AP, Proteintech), anti-pDRP1 (Ser616) (1:1000, 3455, CST), anti-DRP1 (1:1000, 5391, CST), and anti-OPA1 (1:1000, 27733-1-AP, Proteintech). The membranes were washed with TBS-T and incubated with secondary anti-mouse IgG (1:2000, 7076, CST) or anti-rabbit IgG (1:2000, 7074, CST) antibody conjugated to horseradish peroxidase (HRP) for 1 h. Immunoreactive signals were detected with the ECL Western blotting detection reagents (GE Healthcare) and visualized using LAS 4000mini (GE Healthcare). Signal intensities were quantified using ImageQuant (GE Healthcare). All original image data are provided in Supplementary Information 2. Membranes were cut after transfer and prior to antibody incubation to allow the individual detection of target proteins.

### Measurements of mitochondrial OXPHOS complex V activity

Cultured cybrids on a 6-well plate were exposed to ELF-EMF for 0, 6, and 9 h. The cells were homogenized using a glass Dounce homogenizer in MAS buffer (220 mM mannitol, 10 mM KH_2_PO_4_, 5 mM MgCl_2_, 1 mM EGTA, 70 mM sucrose, 2 mM HEPES, and 0.2% (w/v) BSA, pH 7.3). The homogenates were centrifuged at 800 × *g* for 8 min at 4 ˚C. The supernatant was centrifuged again at 10,300 × *g* for 20 min at 4 ˚C to precipitate mitochondria. The Complex V activity of the precipitated mitochondria was measured with the MitoTox Complex V OXPHOS Activity Assay Kit (ab109907, Abcam) and by monitoring the absorbance at 340 nm with BioTek Cytation 5 (Agilent).

### Measurement of oxygen consumption rate (OCR)

Cybrids were seeded at 1 × 10^4^ per well of the Seahorse XFp Cell Culture Miniplate (Agilent) one day prior to measurement. A half of an 8-well plate containing cybrids without ELF-EMF was sandwiched by 1-mm thick Mu-metals (Ohtama), which reduced the magnetic flux intensity from 100 mG (10 µT) to ~ 15 mG (~ 1.5 µT). In contrast, the other half of the plate was exposed to ELF-EMF for 9–12 h.

Cybrids were incubated with the Seahorse XF Base Medium (without phenol red) (Agilent) supplemented with 10 mM glucose, 1 mM sodium pyruvate, 2 mM L-glutamine, 0.4 mM uridine, and 5 mM HEPES-NaOH pH 7.4 for 1 h at 37 °C in a CO_2_-free incubator. The OCR of the cybrids was measured by the Seahorse XFp Extracellular Flux Analyzer (Agilent) according to the manufacturer’s protocols. Oligomycin (1 µM), FCCP (0.7 µM), and rotenone/antimycin A (0.5 µM each) were serially injected into the well, and the OCR was measured for one min three times at each step. The OCR was analyzed by Seahorse Wave Controller Software version 2.6.3 (https://www.agilent.com/en/product/cell-analysis/real-time-cell-metabolic-analysis/xf-software/seahorse-wave-controller-software-2-6-1-740904, Agilent).

### Measurements of ATP level

Cultured cybrids on a 96-well plate were placed on the ELF-EMF device. Every third of a 96-well plate was sandwiched by 1-mm thick Mu-metals for 12, 9, and 0 h to expose cybrids to ELF-EMF for 0, 9, and 12 h, respectively. All cybrids were harvested at 12 h. The cell homogenates were mixed with 100 µl working solution (ATP Assay Kit-Luminescence, 340–09791, Dojindo) on a microplate mixer for 10 min at 25 °C in dark. Luminescent signals were measured by PowerScan MX (DS Pharma Biomedical). ATP levels were calculated by a calibration curve generated by serial dilutions of standard ATP solution with serum-free medium.

### Statistical analysis

Values were represented by mean ± S.D. One-way, ordinary two-way, or repeated measures two-way ANOVA with Dunnett’s post-hoc test, or Student’s *t*-test was performed with GraphPad Prism 10.3.1. *P*-value less than 0.05 was considered statistically significant.

## Electronic supplementary material

Below is the link to the electronic supplementary material.

Supplementary Material 1

Supplementary Material 2

## Data Availability

Data is provided within the manuscript or supplementary information files.

## Abbreviations

ELF-EMF: Extremely low-frequency electromagnetic field
NARP: Neuropathy, ataxia, retinitis pigmentosa
mtDNA: Mitochondrial DNA
mtRNA: Mitochondrial RNA
OXPHOS: Oxidative phosphorylation
ROS: Reactive oxygen species
OCR: Oxygen consumption rate
