# Supplementary material for: Extremely low-frequency electromagnetic field (ELF-EMF) enhances mitochondrial energy production in NARP cybrids
Source: Sci Rep. 2025 Jul 8;15:24369. doi: 10.1038/s41598-025-10536-7 (PMC12238397; doi:10.1038/s41598-025-10536-7)
Supplement: Supplementary file 1 — Supplementary Material 1 [file 41598_2025_10536_MOESM1_ESM.docx]

**Supplementary information 1**

**Supplementary Fig. S1** Comparison of OCRs between 2SA and NARP3-2 cells. The OCR profiles of 2SA and NARP3-2 cybrids were similar without ELF-EMF. The OCR was normalized to that at Phase I. Mean and SD are indicated. **p* < 0.05 by Student’s t test.

**Supplementary Table S1. Primers sequences for qPCR and qRT-PCR**

| **Gene** | **Sequence (5′-3′)** | **Size (bp)** |
| --- | --- | --- |
| Wild-type *MT-ATP6* | CCTACTCATTCAACCAATAGCACt | 186 |
|  | TGGATTAAGGCGACAGCGAT |  |
| m.8993T>G *MT-ATP6* | CCTACTCATTCAACCAATAGCACg | 186 |
|  | TGGATTAAGGCGACAGCGAT |  |
| *MT-ATP8* | ATGGCCCACCATAATTACCC | 170 |
|  | GCAATGAATGAAGCGAACAG |  |
| *ATP5PF* | GGTCAGCCGTCTCAGTCCATT | 153 |
|  | AACTAGCATCAACAGGTCCTC |  |
| *MT-ND6* | CGATTGATGAAAAGGCGGTTG | 112 |
|  | TGATGAAACTTCGGCTCACTCC |  |
| *YWHAZ* | TGCTTGCATCCCACAGACTA  AGGCAGACAATGACAGACCA | 126 |

For allele-specific primers, an artificially introduced mismatch to increase the discrimination power is indicated by underlines, and allele-specific nucleotides are indicated by lowercase letters at the 3´ end.
