# Supplementary material for: Extremely low-frequency electromagnetic field (ELF-EMF) enhances mitochondrial energy production in NARP cybrids
Source: Sci Rep. 2025 Jul 8;15:24369. doi: 10.1038/s41598-025-10536-7 (PMC12238397; doi:10.1038/s41598-025-10536-7)

**Supplementary information 2**

**Original images of Figure 2**The membrane blots were cut after transfer and before antibody incubation for individual detection of target proteins.


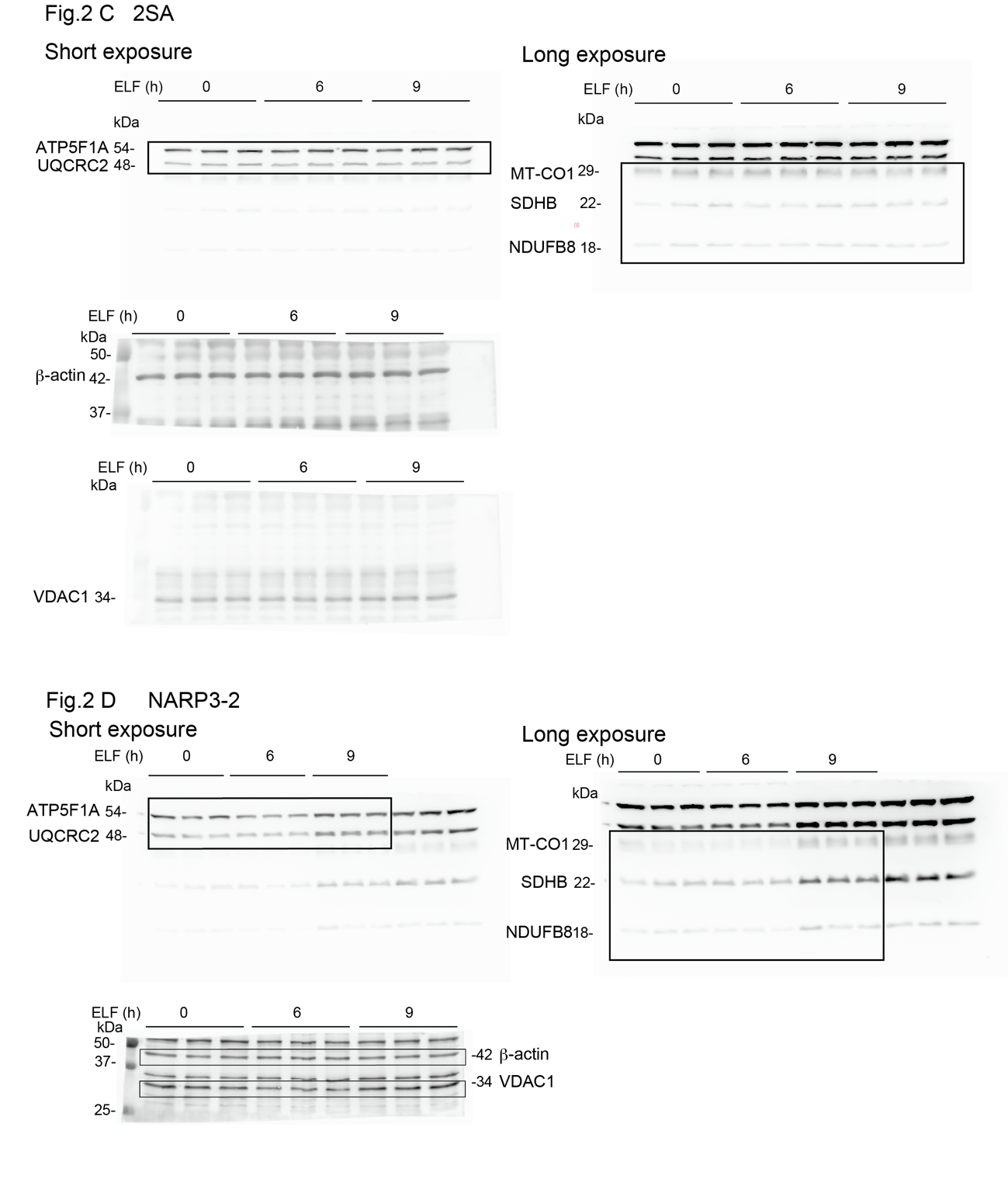


**Original image of Figure 3**

The membrane blots were cut after transfer and before antibody incubation for individual detection of target proteins.


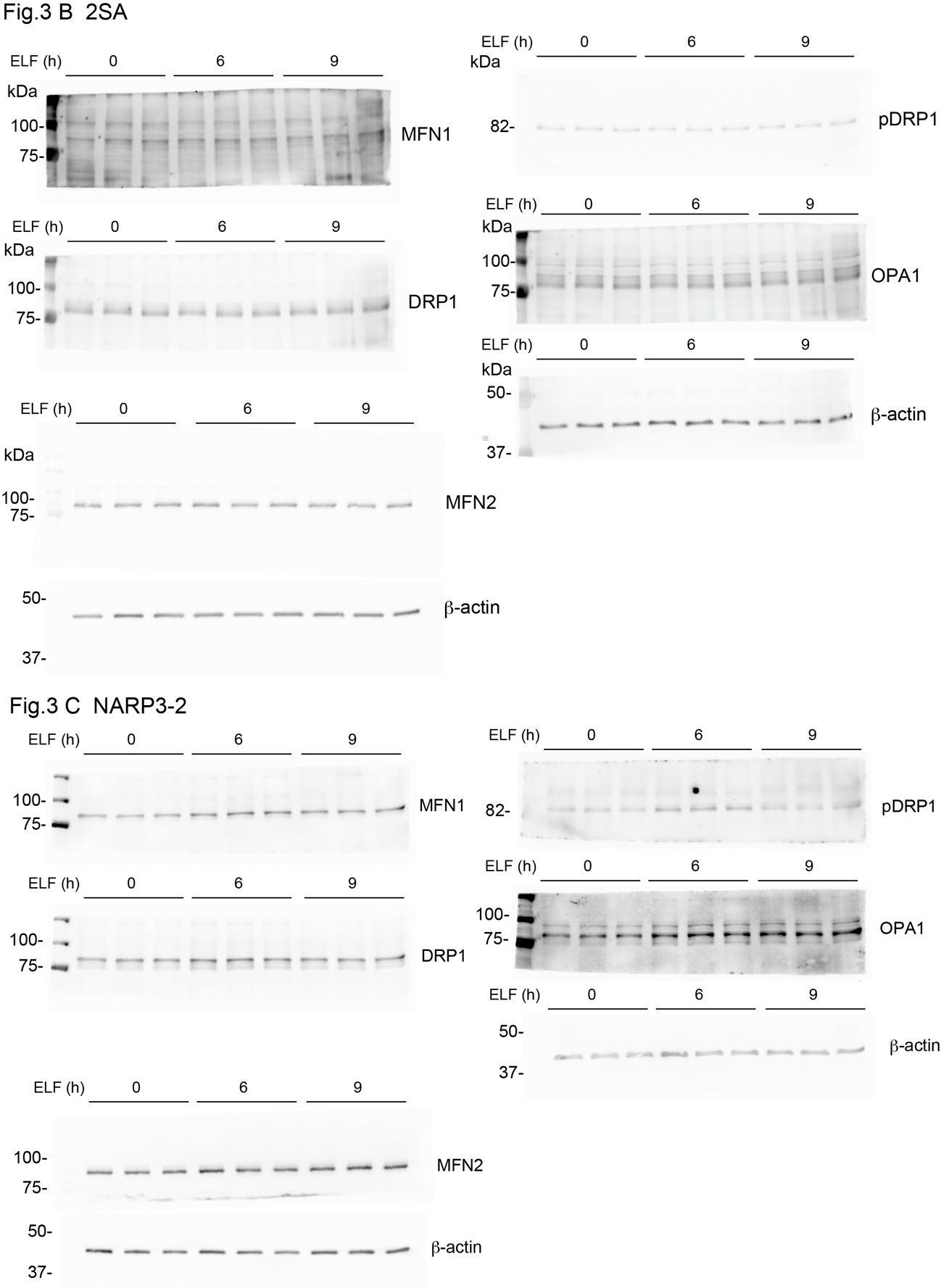

Supplement: Supplementary file 2 — Supplementary Material 2 [file 41598_2025_10536_MOESM2_ESM.docx]
